# Inhibition of UFM1 expression suppresses cancer progression and is linked to the dismal prognosis and immune infiltration in oral squamous cell carcinoma

**DOI:** 10.18632/aging.205219

**Published:** 2023-11-17

**Authors:** Di Ke, Hao-Han Guo, Ni Jiang, Rong-Shu Shi, Teng-Yang Fan

**Affiliations:** 1 Department of Radiology, Affiliated Hospital of Zunyi Medical University, Zunyi, China; 2 Department of Stomatology, Affiliated Hospital of Zunyi Medical University, Zunyi, China; 3 Department of Obstetrics and Gynecology, Women and Children’s Hospital of Chongqing Medical University, Chongqing, China; 4 Department of General Medicine, Affiliated Hospital of Zunyi Medical University, Zunyi, China

**Keywords:** UFM1, OSCC, immune cells, biomarkers, ubiquitination

## Abstract

Background: Ubiquitin fold modifier 1 (UFM1) overexpression is associated with cancer cell proliferation, migration and invasion. However, the roles and pathways of UFM1 in oral squamous cell carcinoma (OSCC) has remained undefined.

Methods: The expression of UFM1 and the relationship between UFM1 expression and prognosis were investigated using data of OSCC patients from The Cancer Genome Atlas (TCGA) database. The UFM1 co-expressed genes, and the association between the UFM1 expression and immune cells and ubiquitination were explored. The effects of UFM1 expression on the growth and migration of OSCC cells were investigated by siRNA interference, Cell Counting Kit-8 (CCK-8), Transwell, Western blotting, and wound healing experiments.

Results: UFM1 was highly expressed in OSCC. UFM1 overexpression was associated with short overall survival, disease-specific survival, and progression-free interval, and was an adverse factor for prognosis in OSCC. UFM1-related nomograms were significantly associated with poor prognosis in OSCC patients. Decreased UFM1 expression could inhibit the proliferation, migration, and invasion of OSCC cells. UFM1 was associated with the immune cells (such as the Th17 cells, T helper cells, and cytotoxic cells) and ubiquitination.

Conclusion: Elevated UFM1 expression was associated with poor prognosis, ubiquitination and immune infiltration in OSCC, and inhibition of UFM1 expression delayed OSCC progression, showing that UFM1 could be a biomarker for prognosis and treating OSCC patients.

## INTRODUCTION

Oral squamous cell carcinoma (OSCC) is one of the malignancies. The incidence of OSCC has remained elevated in recent years. Studies have demonstrated that some biomarkers are associated with the prognosis of OSCC patients [1–5]. However, due to the lacking specific diagnostic biomarkers and effective therapeutic targets in the early stages of OSCC patients, the prognosis remained poor. Therefore, it is critical to find new targets to improve the survival time of OSCC patients.

Ubiquitin Fold Modifier 1 (UFM1) is a ubiquitin-like protein that could be coupled to the target protein like ubiquitination through the E1-like activating enzyme UBA5 and E2-like coupling enzyme UFC1 [6]. Previous studies confirmed that the changes in UFM1 expression were related to the progression of gastric cancer (GC), hepatocellular carcinoma (HCC), and breast cancer [7–10]. For example, the levels of UFM1 were down-regulated in GC tissues. Decreased UFM1 expression was associated with 5-year survival in GC patients. Increased UFM1 expression could reduce the invasion and migration ability of GC AGS and HGC-27 cells, while downregulation of UFM1 expression could promote the invasion and migration of GC cells. UFM1 could increase the ubiquitination levels of pyruvate dehydrogenase kinase 1 (PDK1), and inhibit the expression of PDK1 protein, thereby inhibiting the phosphorylation level of AKT serine/threonine kinase 1 (AKT) at Ser473 [7]. Long strand non-coding RNA B3GALT5-AS1 expression was significantly decreased in HCC. B3GALT5-AS1 overexpression could delay the malignant features of HCC HCCLM3 cells. UFM1 overexpression could reduce the invasion and migration ability in HCC HCCLM3 cells, indicating that B3GALT5-AS1 could inhibit the HCC progression by promoting UFM1 expression [8]. Currently, the roles and mechanisms of UFM1 in OSCC have not been reported. Therefore, we aimed to identify the role of UFM1 in OSCC via data analysis and basic research and explore the relationship between the expression levels of UFM1 and the immune infiltrating cells and ubiquitination in OSCC to provide a new candidate marker for OSCC patients.

## MATERIALS AND METHODS

### Data sources and identification of UFM1 gene expression

In August 2022, the UFM1 gene expression data of 32 normal tissues and 329 primary OSCC tissues were downloaded from the Cancer Genome Atlas (TCGA (https://www.cancer.gov/TCGA)) database, covering both data types: the reads per kilobase of transcript per million reads mapped (FPKM) and transcripts per million (TPM). The UFM1 gene expression data in normal and OSCC tissues were extracted using Perl language. Subsequently, the expression levels of UFM1 in normal and OSCC tissues were identified. In addition, the data from normal tissues and cancer tissues were matched and sorted. It was found that 32 patients had the normal tissues and matched cancer tissues. The expression levels of UFM1 in normal and cancer tissues from 32 paired cancer patients were identified.

### Prognostic values of UFM1

The UFM1 gene expression data were matched to the data of overall survival (OS), disease-specific survival (DSS) and progression-free interval (PFI) of cancer patients into groups. Dichotomous grouping was performed via the UFM1 expression level, and survival analysis was used to investigate the relationship between UFM1 expression and the OS, DSS, and cancer progression in OSCC patients.

### The roles of UFM1 expression in the prognosis of subgroup patients with OSCC

In the OS, DSS, and disease progression of OSCC patients, cancer patients were divided into subgroups according to the T stage, lymph node metastasis (N stage) and distant metastasis (M stage). The affiliation between UFM1 expression and OS, DSS, and disease progression in OSCC patients was identified through survival analysis.

### Relationship between UFM1 expression and clinical characteristics of OSCC patients

The expression levels of UFM1 in the tissues of deceased and surviving patients were identified under OS, DSS, and disease progression in OSCC patients. UFM1 expression was used for the classification. Moreover, the chi-square test was conducted to identify the relationship between the UFM1 expression and T stage, N stage, M stage, clinical stage, radiotherapy treatment, age, gender, tissue grade, smoking history, and OS, DSS, and PFI endpoint events in OSCC patients.

### Cox regression analysis and construction of nomogram

Univariate Cox regression analysis was performed on T stage (T1, T2, T3 and T4), N stage (N0, N1, N2 and N3), M stage (M0 and M1), and UFM1 (overexpression and low expression) and association among OSCC patients and OS, DSS, and disease progression. Furthermore, multivariate Cox regression analysis was executed based on P < 0.05. In addition, the prognostic nomograms of T stage, N stage, M stage and UFM1 expression and nomogram-related visualizations were constructed based multivariate Cox regression analysis by rms package in R language.

### The pathways of UFM1 co-expressed genes

UFM1 co-expressed genes were obtained by correlation analysis and defined as UFM1 strongly co-expressed genes based on the absolute value of the coefficient of 0.4 and the P-value less than 0.001. KEGG analysis could explore the pathways involved in multiple genes. Therefore, we utilized KEGG analysis to analyze the signaling pathways involved in UFM1 co-expressed genes in the DAVID (https://david.ncifcrf.gov/) database [11].

### Analysis of the relationship between UFM1 expression levels and immune infiltrating cells

The immune cells of OSCC tissues were scored by single sample gene set enrichment analysis (GSEA) technology, and the Pearson correlation was used to analyze the relationship between the expression levels of UFM1 and 24 types of immune cells (NK CD56bright cells, aDC, Th2 cells, DC, T cells, iDC, Tem, mast cells, TReg, B cells, NK cells, T helper cells, pDC, macrophages, Tcm, neutrophils, TFH, Tgd, CD8 T cells, Th1 cells, cytotoxic cells, Th17 cells, T helper cells, and NK CD56dim cells) levels, and the significantly correlated immune cells were obtained based on the P<0.05.

### Identification of the relationship between UFM1 expression and ubiquitination

The ubiquitination was entered into on the official website of GSEA (https://www.gsea-msigdb.org/gsea/) and the histone ubiquitination gene set was selected [12]. Correlation analysis between UFM1 expression and histone ubiquitination genes PCGF3, LEO1, DTX3L, DDB1, DDB2, RNF168, RYBP, UHRF1, TRIM37, WAC, PAF1, BCOR, RNF20, ATXN7L3, RAG1, RING1, RNF2, BMI1, SKP1, UBE2E1, PCGF2, CDC73, PCGF6, PCGF5, CUL4B, KDM2B, PCGF1, CTR9, and RNF40 expression levels were conducted, and the significantly correlated ubiquitination genes were obtained based on the P<0.05.

### Cell culture and transfection

OSCC cells (cal27) were donated by laboratory researchers in the Affiliated Hospital of Zunyi Medical University. The cells were cultured in DMEM medium with 10% fetal bovine serum. UFM1 expression levels were interfered with by siRNA technology, cells were in a good state of growth and culture, and the density was suitable [13]. The siRNA target sequence for UFM1 was CCTGCTGCAACAAGTGCAATT. Total RNA and proteins from the control group (NC) and UFM1 expression inhibition group (si-UFM1) were collected 24 h after transfection to verify the success of the cell model and conduct subsequent cell function studies.

### Identification of UFM1 expression in the cell model

Total cell RNAs were transcribed, and the Polymerase Chain Reaction (PCR) was performed according to standard procedure [13]. UFM1 expression levels in both cell groups were then calculated via the 2^−ΔΔCt^ formula. The PCR primers of UFM1 were: forward 5’-TCGGAAGTGCTGATGAGTT-3’ and reverse 5’-CCTCCTTAATA GAAGCCTGGT-3’. The collected proteins were subjected to BCA quantification, protein denaturation, electrophoresis, electrolysis, membrane washing, incubation of 1:1000 UFM1 antibody (Abcam, UK), secondary antibody incubation, and protein exposure.

### Cell proliferation

After the transfection of cal27 cells, 96-well plates were laid, and the number of cal27 cells in each hole was 2500. Furthermore, the proliferation ability of the cal27 cells in the control group and the interfered with the UFM1 expression group was detected by using the Cell Counting Kit-8 (CCK-8) assay. After adding 10 μL CCK-8 solution to each well and incubating at 37° C and 5% CO_2_ for 2 h, the cell activity was detected using an enzyme marker.

### Cell migration

In the control group and interfered with the UFM1 expression group, the straight line in the six-hole plate was drawn using a 200 μL gun head. The suspended cal27 cells were then washed with phosphate buffers, and then cal27 cells were fed in serum-free medium at 37° C and 5% CO_2_ and photographed at 0 h after the scratch. The cell migration distance was observed 24 and 48 h after the scratch, and photographs were taken when significant differences were reached. Finally, the migration distance of the two groups of cal27 cells was calculated.

### Cell invasion

The transfected cal27 cells were digested and suspended in a serum-free medium. After the cell count, the concentration was adjusted to 1×10^5^/mL. Diluted Matrigel glue was evenly applied to the surface of the film. Moreover, 800 μL medium containing 10% serum was added to the lower chamber of Transwell, 200 μL cell suspension was added to the upper chamber of Transwell, and cultured in a cell culture incubator for 24 h. Cell counts were performed after cell fixation and staining.

### Statistical analysis

Wilcoxon rank-sum and chi-square tests were used to assess the expression of UFM1 in OSCC tissues and to explore whether there was statistical significance between UFM1 expression and clinicopathological characteristics of cancer patients. Survival analysis was used to understand the affiliation between UFM1 overexpression and poor prognosis in OSCC patients. The role of UFM1 expression on proliferation, migration, and invasion was tested using the t-test. Correlation analysis coefficients represented gene-to-gene relationships, and UFM1 expression levels and immune cells. The significance threshold is based on P < 0.05, which was considered significant.

### Availability of data and materials

The data generated during this study are available upon request from the corresponding authors.

## RESULTS

### UFM1 overexpression in OSCC was associated with poor prognosis

**Figure 1:**
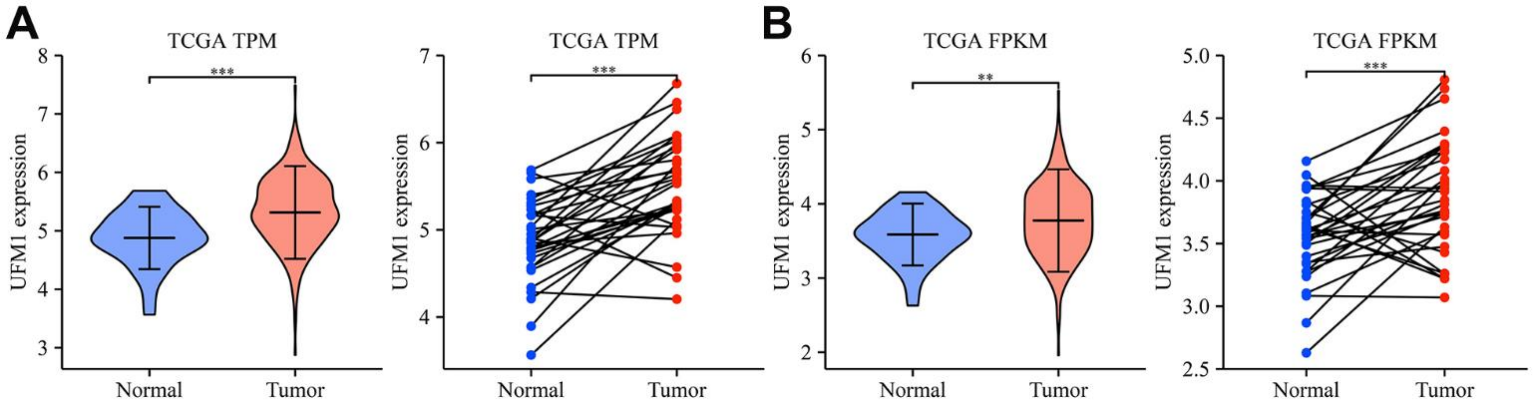
**UFM1 expression significantly increased in OSCC tissues of the TCGA database.** (**A**) The data of TPM type in TCGA database; (**B**) The data of FPKM type in TCGA database. Note: OSCC, oral squamous cell carcinoma; TCGA, The Cancer Genome Atlas; FPKM, reads per kilobase of transcript per million reads mapped; TPM, transcripts per million.

**Figure 2:**
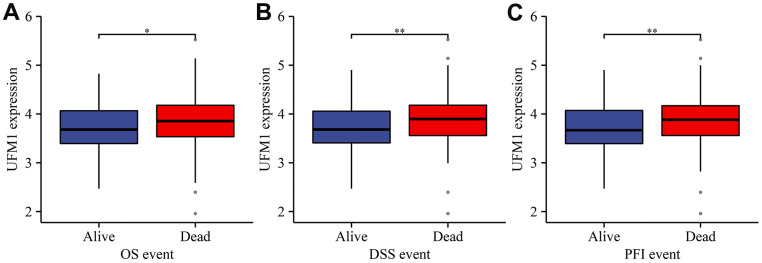
**The relationship between UFM1 expression and dismal prognosis was explored based on the status of cancer patients in TCGA database.** (**A**) OS; (**B**) DSS; (**C**) PFI. Note: OS, overall survival; DSS, disease-specific survival; PFI, progression-free interval.

**Figure 3:**
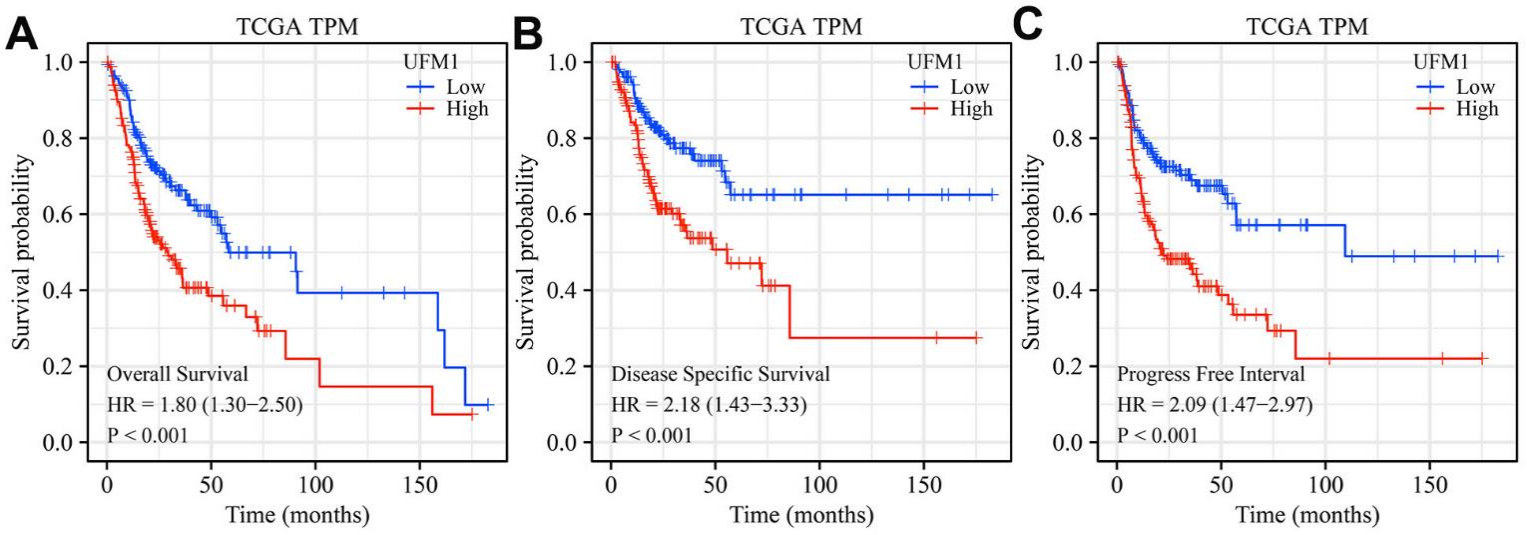
**Elevated UFM1 expression levels associated with dismal prognosis in OSCC patients based on the data of TPM type in TCGA database.** (**A**) OS; (**B**) DSS; (**C**) PFI. Note: OSCC, oral squamous cell carcinoma; TPM, transcripts per million; OS, overall survival; DSS, disease-specific survival; PFI, progression-free interval; TCGA, The Cancer Genome Atlas.

Compared with normal tissues, the expression of UFM1 was significantly enhanced in unpaired and paired tissues (Figure 1A, 1B). Regarding the OS, DSS, and PFI endpoints, UFM1 expression levels were significantly elevated in the tissues of deceased OSCC patients (Figure 2A–2C). Survival analysis displayed that elevated UFM1 expression levels were significantly associated with poor prognostic indicators (OS, DSS, and PFI) in OSCC patients (Figure 3A–3C). Grouping by high- and low-UFM1 expression revealed that UFM1 expression was associated with the OS, DSS, and PFI in OSCC patients (Table 1).

**Table 1:** The relationship between UFM1 expression and clinical characteristics of OSCC patients.

**Characteristic**	**Low UFM1 expression**	**High UFM1 expression**	**P**
N	164	165	
T stage			0.174
T1	11 (3.4%)	7 (2.2%)	
T2	56 (17.6%)	49 (15.4%)	
T3	45 (14.1%)	37 (11.6%)	
T4	48 (15%)	66 (20.7%)	
N stage			0.930
N0	86 (27.3%)	82 (26%)	
N1	27 (8.6%)	29 (9.2%)	
N2	43 (13.7%)	45 (14.3%)	
N3	2 (0.6%)	1 (0.3%)	
M stage			1.000
M0	157 (50.3%)	153 (49%)	
M1	1 (0.3%)	1 (0.3%)	
Clinical stage			0.190
Stage I	6 (1.9%)	5 (1.6%)	
Stage II	39 (12.2%)	40 (12.5%)	
Stage III	40 (12.5%)	25 (7.8%)	
Stage IV	75 (23.5%)	89 (27.9%)	
Gender			0.934
Female	50 (15.2%)	52 (15.8%)	
Male	114 (34.7%)	113 (34.3%)	
Age			0.917
<=60	78 (23.8%)	77 (23.5%)	
>60	85 (25.9%)	88 (26.8%)	
Histologic grade			0.161
G1	31 (9.7%)	21 (6.5%)	
G2	102 (31.8%)	98 (30.5%)	
G3	27 (8.4%)	40 (12.5%)	
G4	1 (0.3%)	1 (0.3%)	
OS event			0.013
Alive	101 (30.7%)	78 (23.7%)	
Dead	63 (19.1%)	87 (26.4%)	
DSS event			0.003
Alive	122 (39.1%)	97 (31.1%)	
Dead	34 (10.9%)	59 (18.9%)	
PFI event			< 0.001
Alive	112 (34%)	82 (24.9%)	
Dead	52 (15.8%)	83 (25.2%)	

Note: OSCC, oral squamous cell carcinoma.

### Elevated UFM1 expression levels were associated with poor prognosis in OSCC patients based on TNM stage

**Figure 4:**
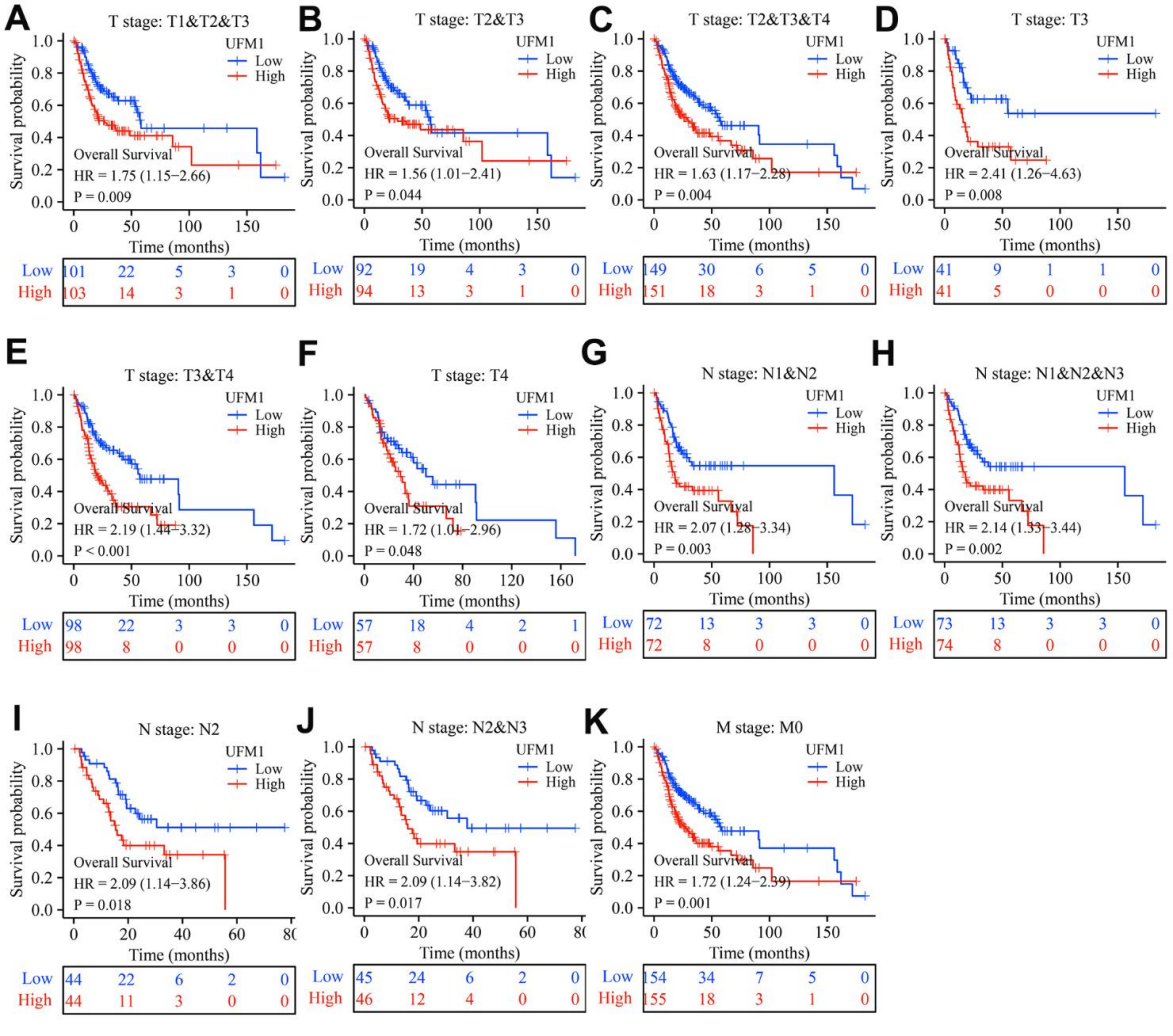
**Elevated UFM1 expression associated with the shorter OS in OSCC patients based on the data of TPM type in TCGA database.** (**A**) Stage T1-3; (**B**) Stage T2-3; (**C**) Stage T2-4; (**D**) Stage T3; (**E**) Stage T3-4; (**F**) Stage T4; (**G**) N1-2; (**H**) N1-3; (**I**) N2; (**J**) N2-3; (**K**) M0. Note: OSCC, oral squamous cell carcinoma; OS, overall survival; TPM, transcripts per million; TCGA, The Cancer Genome Atlas.

**Figure 5:**
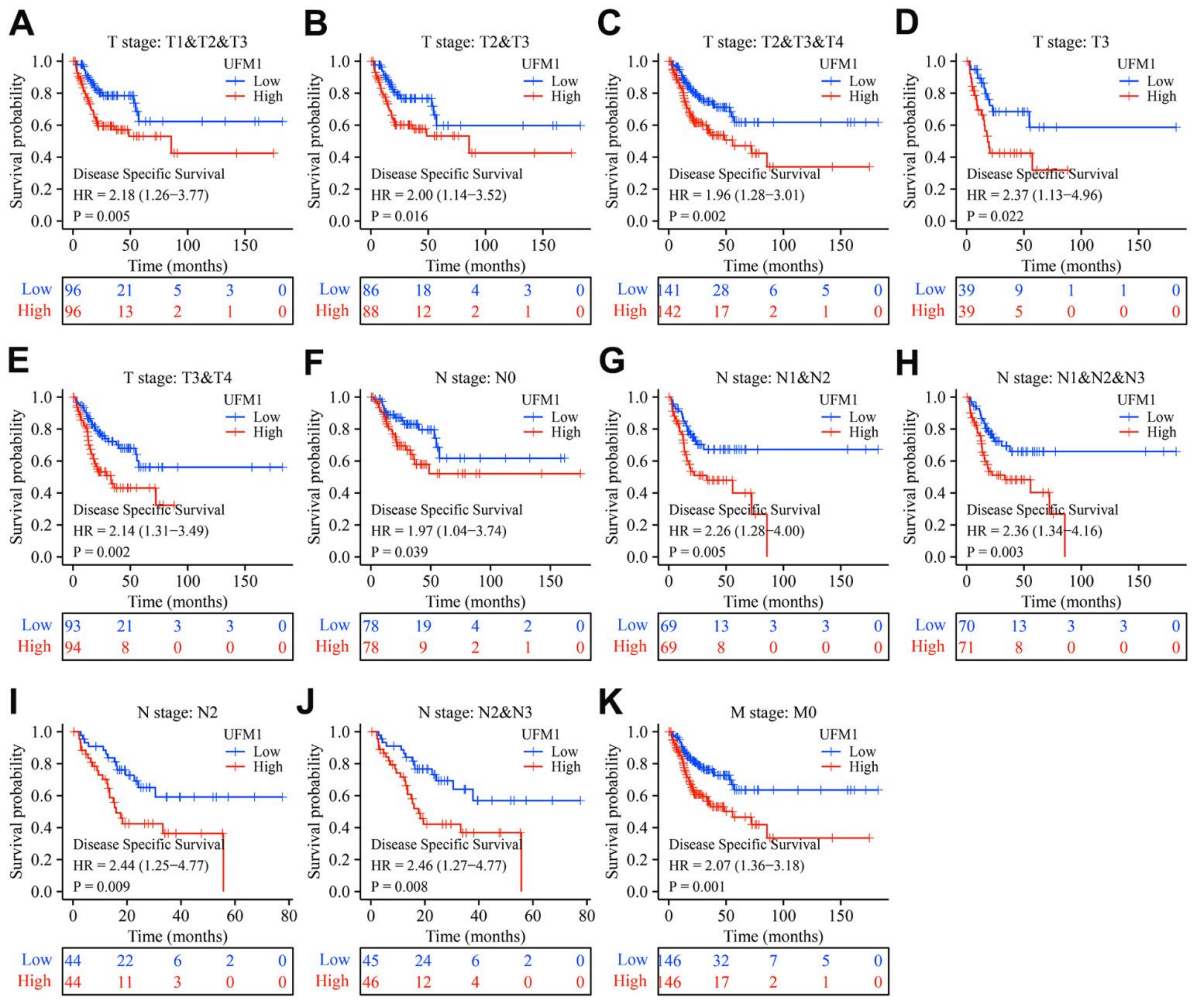
**Elevated UFM1 expression associated with the shorter DSS in OSCC patients based on the data of TPM type in TCGA database.** (**A**) Stage T1-3; (**B**) Stage T2-3; (**C**) Stage T2-4; (**D**) Stage T3; (**E**) Stage T3-4; (**F**) N0; (**G**) N1-2; (**H**) N1-3; (**I**) N2; (**J**) N2-3; (**K**) M0. Note: OSCC, oral squamous cell carcinoma; DSS, disease-specific survival; TPM, transcripts per million; TCGA, The Cancer Genome Atlas.

**Figure 6:**
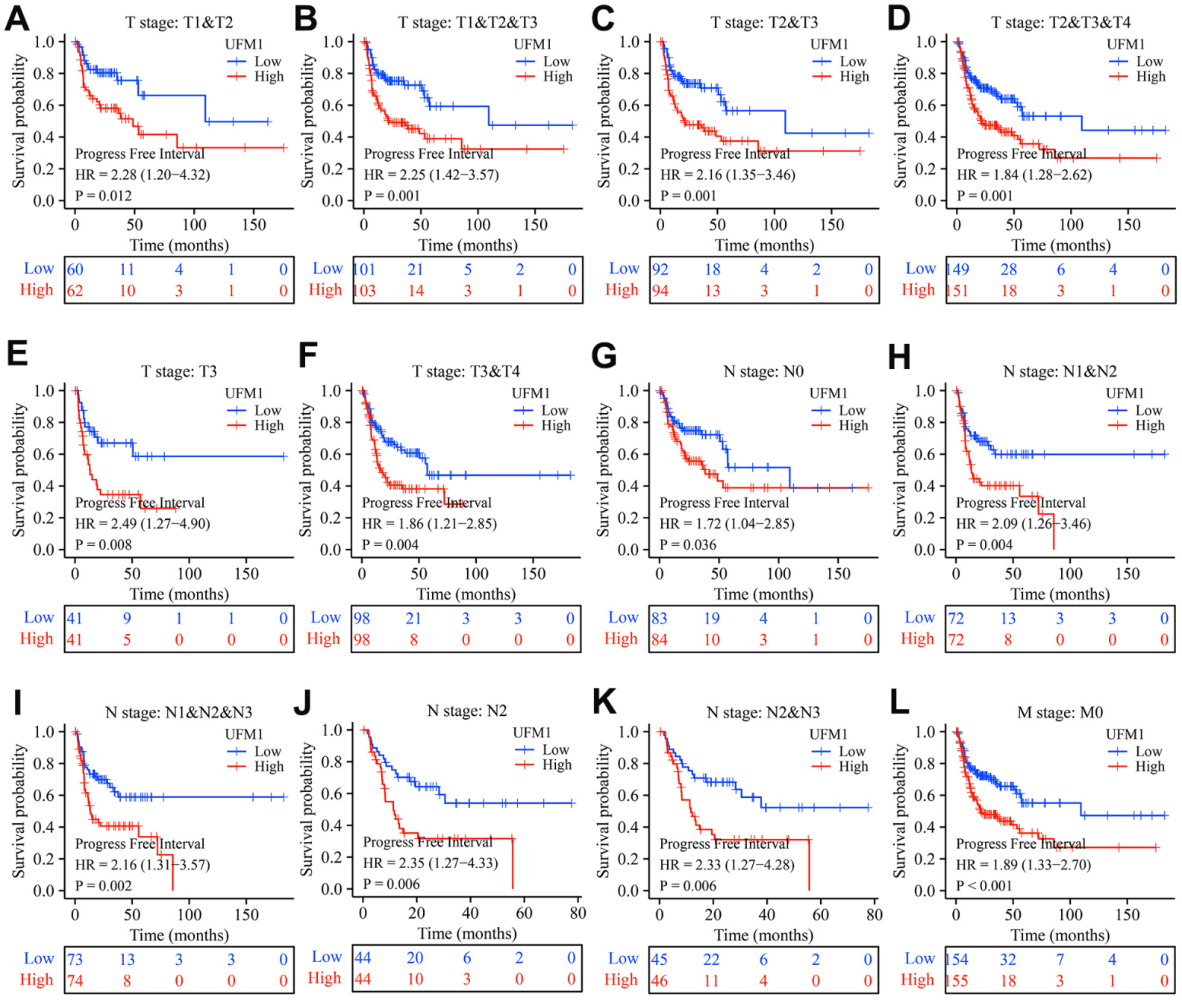
**Elevated UFM1 expression associated with the shorter PFI in OSCC patients based on the data of TPM type in TCGA database.** (**A**) Stage T1-2; (**B**) Stage T1-3; (**C**) Stage T2-3; (**D**) Stage T2-4; (**E**) Stage T3; (**F**) Stage T3-4; (**G**) N0; (**H**) N1-2; (**I**) N1-3; (**J**) N2; (**K**) N2-3; (**L**) M0. Note: OSCC, oral squamous cell carcinoma; PFI, progression-free interval; TPM, transcripts per million; TCGA, The Cancer Genome Atlas.

In patients with OSCC of T1-3, T2-3, T2-4, T3, T3-4, T4, N1-2, N1-3, N2, N2-3, and M0 stages, elevated UFM1 expression was significantly associated with poor survival time in cancer patients (Figure 4). In patients with OSCC of T1-3, T2-3, T2-4, T3, T3-4, N0, N1-2, N1-3, N2, N2-3, and M0 stages, elevated UFM1 expression was significantly associated with the poorer DSS in cancer patients (Figure 5). In patients with OSCC of T1-2, T1-3, T2-3, T2-4, T3, T3-4, N0, N1-2, N1-3, N2, N2-3 and M0 stages, elevated UFM1 expression was significantly associated with the poorer PFI in cancer patients (Figure 6).

### Nomograms of UFM1

**Figure 7:**
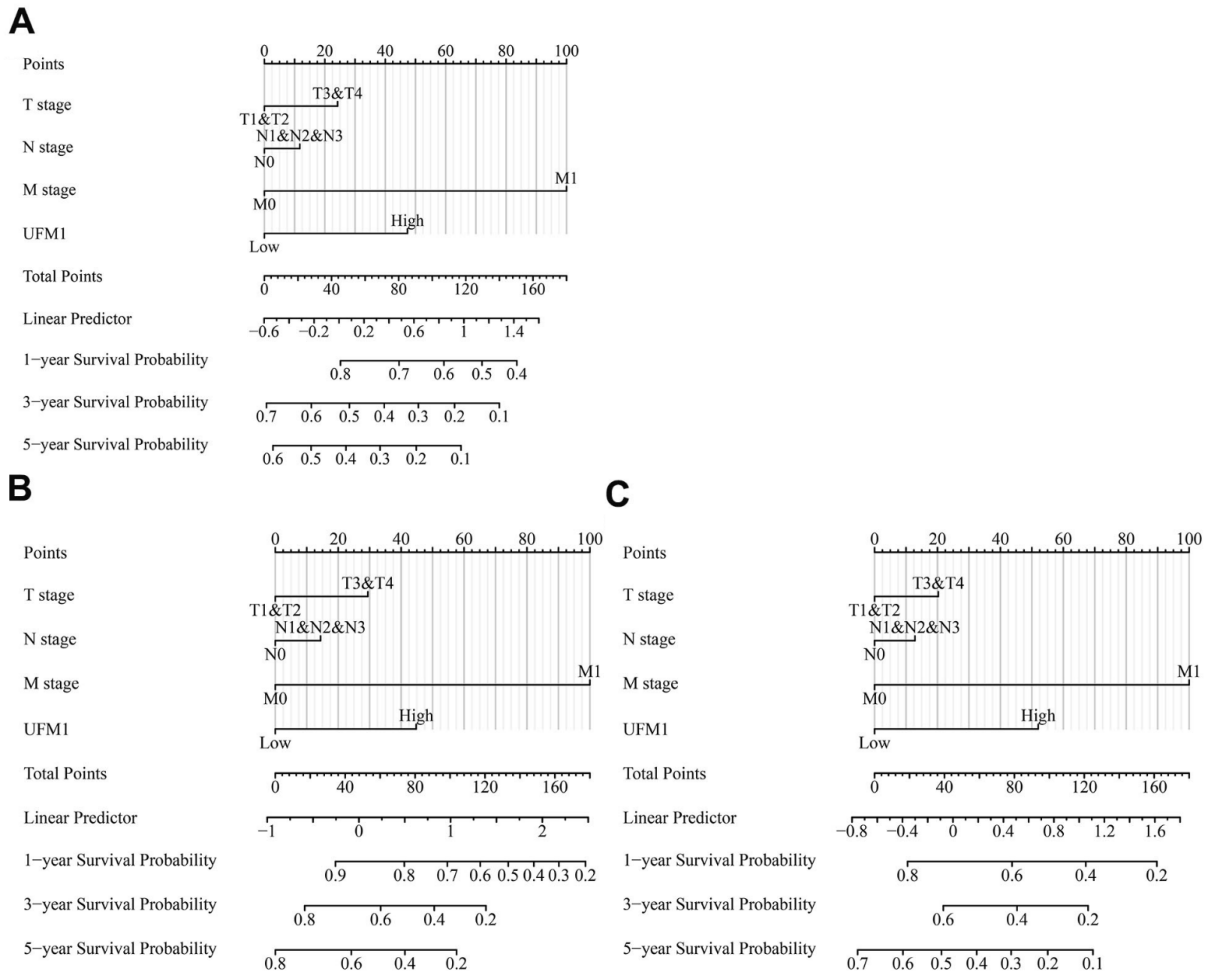
**The OS, DSS and PFI-related nomograms based on the T, N, and M stages and UFM1 expression based on the data of TPM type in TCGA database.** (**A**) OS; (**B**) DSS; (**C**) PFI. Note: OS, overall survival; DSS, disease-specific survival; PFI, progression-free interval; TPM, transcripts per million; TCGA, The Cancer Genome Atlas.

Univariate Cox regression analysis depicted that UFM1 overexpression was an influencing factor of poor prognosis indicators OS, DSS and PFI in OSCC patients (Tables 2–4). In addition, N stage was also an influencing factor for DSS and PFI in OSCC patients (Tables 3, 4). T, N, and M stages were risk factors for dismal prognosis in OSCC patients. Therefore, we constructed the nomograms of the T, N, and M stages and UFM1 expression levels (Figure 7).

**Table 2:** The factors affecting prognostic indicator overall survival by univariate Cox regression analysis.

**Characteristics**	**N**	**HR (95% CI)**	**P**
T stage	318		
T1	18	Reference	
T2	104	1.056 (0.475-2.349)	0.893
T3	82	1.511 (0.676-3.378)	0.315
T4	114	1.378 (0.629-3.020)	0.423
N stage	314		
N0	167	Reference	
N1	56	1.131 (0.737-1.735)	0.574
N2&N3	91	1.493 (1.022-2.180)	0.038
M stage	311		
M0	309	Reference	
M1	2	2.621 (0.365-18.838)	0.338
UFM1 expression	328		
Low	163	Reference	
High	165	1.770 (1.277-2.454)	<0.001

**Table 3:** The factors affecting prognostic indicator DSS by univariate Cox regression analysis.

**Characteristics**	**N**	**HR (95% CI)**	**P**
T stage	301		
T1	18	Reference	
T2	96	0.998 (0.342-2.908)	0.996
T3	78	2.101 (0.741-5.959)	0.163
T4	109	1.587 (0.565-4.454)	0.381
N stage	297		
N0	156	Reference	
N1	50	1.013 (0.550-1.866)	0.966
N2&N3	91	2.116 (1.351-3.314)	0.001
M stage	294		
M0	292	Reference	
M1	2	3.773 (0.522-27.287)	0.188
UFM1 expression	311		
Low	155	Reference	
High	156	2.150 (1.408-3.282)	<0.001

Note: DSS, disease-specific survival.

**Table 4:** The factors affecting prognostic indicator PFI by univariate Cox regression analysis.

**Characteristics**	**N**	**HR (95% CI)**	**P**
T stage	318		
T1	18	Reference	
T2	104	1.697 (0.606-4.750)	0.314
T3	82	2.402 (0.855-6.744)	0.096
T4	114	2.129 (0.769-5.894)	0.146
N stage	314		
N0	167	Reference	
N1	56	0.975 (0.594-1.599)	0.919
N2&N3	91	1.649 (1.121-2.425)	0.011
M stage	311		
M0	309	Reference	
M1	2	2.524 (0.352-18.116)	0.357
UFM1 expression	328		
Low	163	Reference	
High	165	1.947 (1.375-2.757)	<0.001

Note: OPFI, progression-free interval.

### Pathways of UFM1 co-expressed genes

**Figure 8:**
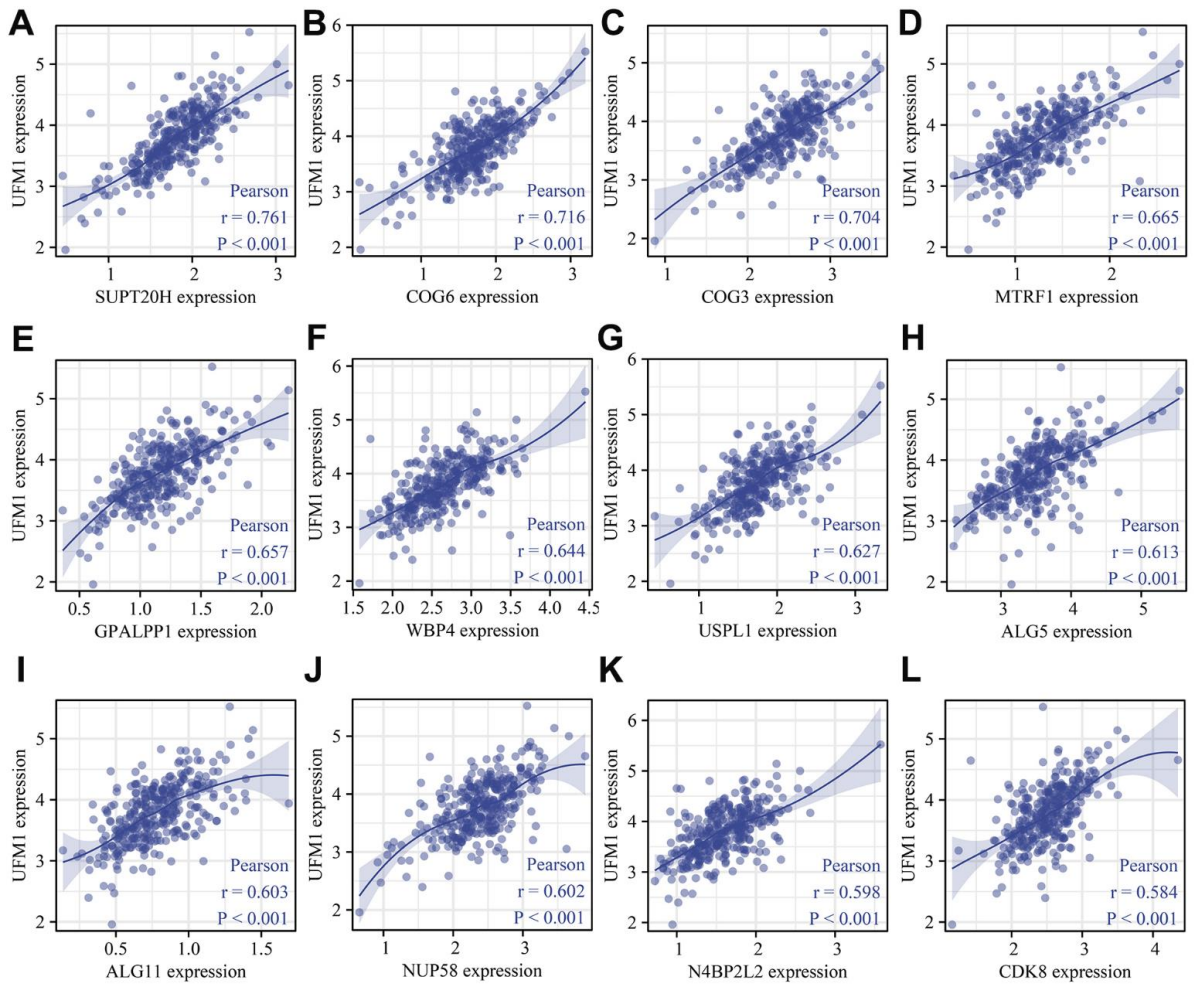
**UFM1 co-expressed genes were shown using scatter plot.** (**A**) SUPT20H; (**B**) COG6; (**C**) COG3; (**D**) MTRF1; (**E**) GPALPP1; (**F**) WBP4; (**G**) USPL1; (**H**) ALG5; (**I**) ALG11; (**J**) NUP58; (**K**) N4BP2L2; (**L**) CDK8.

There were 148 UFM1 positively correlated co-expressed genes (Table 5). In the David database, KEGG analysis showed that UFM1 co-expressed genes involved the autophagy, Biosynthesis of nucleotide sugars, Metabolic pathways, Amino sugar and nucleotide sugar metabolism, and N-Glycan biosynthesis B signal mechanism (P <0.05). In addition, Figure 8 showed that UFM1 was significantly correlated with the expression levels of SUPT20H, COG6, COG3, MTRF1, GPALPP1, WBP4, USPL1, ALG5, ALG11, NUP58, N4BP2L2, and CDK8.

**Table 5:** UFM1 co-expressed genes.

**Gene**	**Cor**	**Gene**	**Cor**	**Gene**	**Cor**	**Gene**	**Cor**
SUPT20H	0.761	SEPTIN7	0.483	ODR4	0.450	TRMT11	0.412
COG6	0.716	NAA16	0.483	CKAP2	0.445	TRMT13	0.412
COG3	0.704	RFC3	0.482	IMPA1	0.443	PCNX4	0.411
MTRF1	0.665	EXOSC8	0.481	SLC25A15	0.442	TMED2	0.410
GPALPP1	0.657	SLC25A30	0.479	POT1	0.437	TARS1	0.410
WBP4	0.644	PROSER1	0.479	SBDSP1	0.437	LACC1	0.410
USPL1	0.627	NUDT15	0.477	SNX14	0.437	DCAF17	0.410
ALG5	0.613	MICU2	0.476	KATNAL1	0.436	BBIP1	0.409
ALG11	0.603	NEK3	0.474	ZDHHC17	0.433	MPHOSPH8	0.409
NUP58	0.602	CCDC122	0.473	PCID2	0.433	SPCS3	0.409
N4BP2L2	0.598	HMGB1	0.472	DNAJC24	0.431	DPM1	0.409
CDK8	0.584	VWA8	0.468	STARD3NL	0.431	SNX6	0.409
NUFIP1	0.570	ELF1	0.468	ARF4	0.431	SELENOF	0.409
MRPS31	0.549	OBI1	0.4681	ZRANB2	0.429	RAP2A	0.408
ESD	0.544	MZT1	0.465	MAP3K7	0.427	TM9SF2	0.407
LRCH1	0.538	CDADC1	0.464	CAPZA2	0.426	AKAP10	0.407
FNDC3A	0.537	VPS36	0.464	FAM76B	0.426	PRELID3B	0.407
UGGT2	0.532	CCDC82	0.464	USP12	0.426	STAM2	0.407
MTMR6	0.526	PIBF1	0.464	ABCE1	0.426	AL390728.4	0.407
NHLRC3	0.526	MRPS31P4	0.463	GLS	0.424	ZMYM2	0.407
SPRYD7	0.521	TMCO3	0.462	DPY19L4	0.423	GOLGA5	0.407
MED4	0.517	PAN3	0.462	EIF2A	0.423	GNPDA2	0.406
RFXAP	0.514	BFAR	0.461	SLC39A10	0.419	PGM3	0.405
GTF2F2	0.513	IFT88	0.461	RPE	0.419	MTRR	0.404
INTS6	0.509	TMEM87B	0.460	C2orf49	0.416	HMGN4	0.404
RCBTB1	0.509	KBTBD6	0.459	SETDB2	0.416	XRN2	0.404
RNF6	0.504	COPB1	0.459	SUCLA2	0.416	ZNF143	0.404
UTP14C	0.502	PRKAA1	0.458	TAF1A	0.415	STX2	0.404
TGDS	0.502	TPP2	0.458	CDC16	0.414	PCNP	0.403
PDS5B	0.499	DNAJB14	0.456	POLR1D	0.414	SLC25A32	0.403
ZMYM5	0.499	GPR180	0.455	ZCCHC4	0.414	TMED5	0.402
KPNA3	0.498	LRRC40	0.455	NUDCD1	0.414	FBXL2	0.401
DGKH	0.495	PMM2	0.454	RBM18	0.413	GOPC	0.401
AKAP11	0.495	RB1	0.452	GTF2H1	0.413	TMEM267	0.401
TANK	0.488	ZC3H7A	0.452	IPO5	0.412	ZDHHC20	0.401
ABHD13	0.487	SUGT1	0.452	SNRNP27	0.412	CUL4A	0.400
ZC3H13	0.4858	TES	0.452	BBS7	0.412	UFM1	1.00

### Decreased UFM1 expression inhibited the growth and migration of OSCC cells

**Figure 9:**
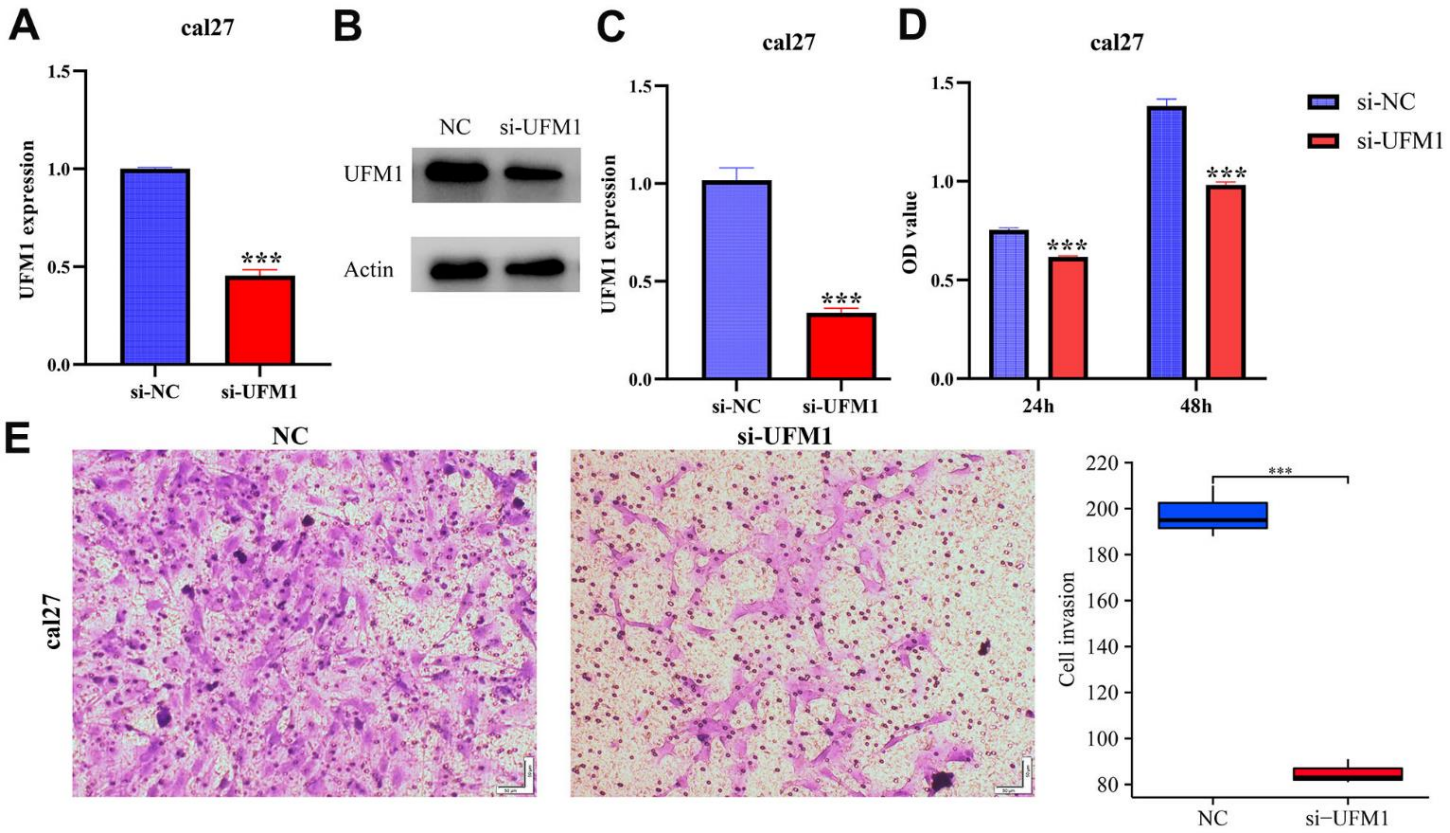
**Inhibiting UFM1 expression significantly reduces cell proliferation and invasion via CCK-8 and Transwell.** (**A**–**C**) Establishment of cell model; (**D**) Cell proliferation; (**E**) Cell invasion.

**Figure 10:**
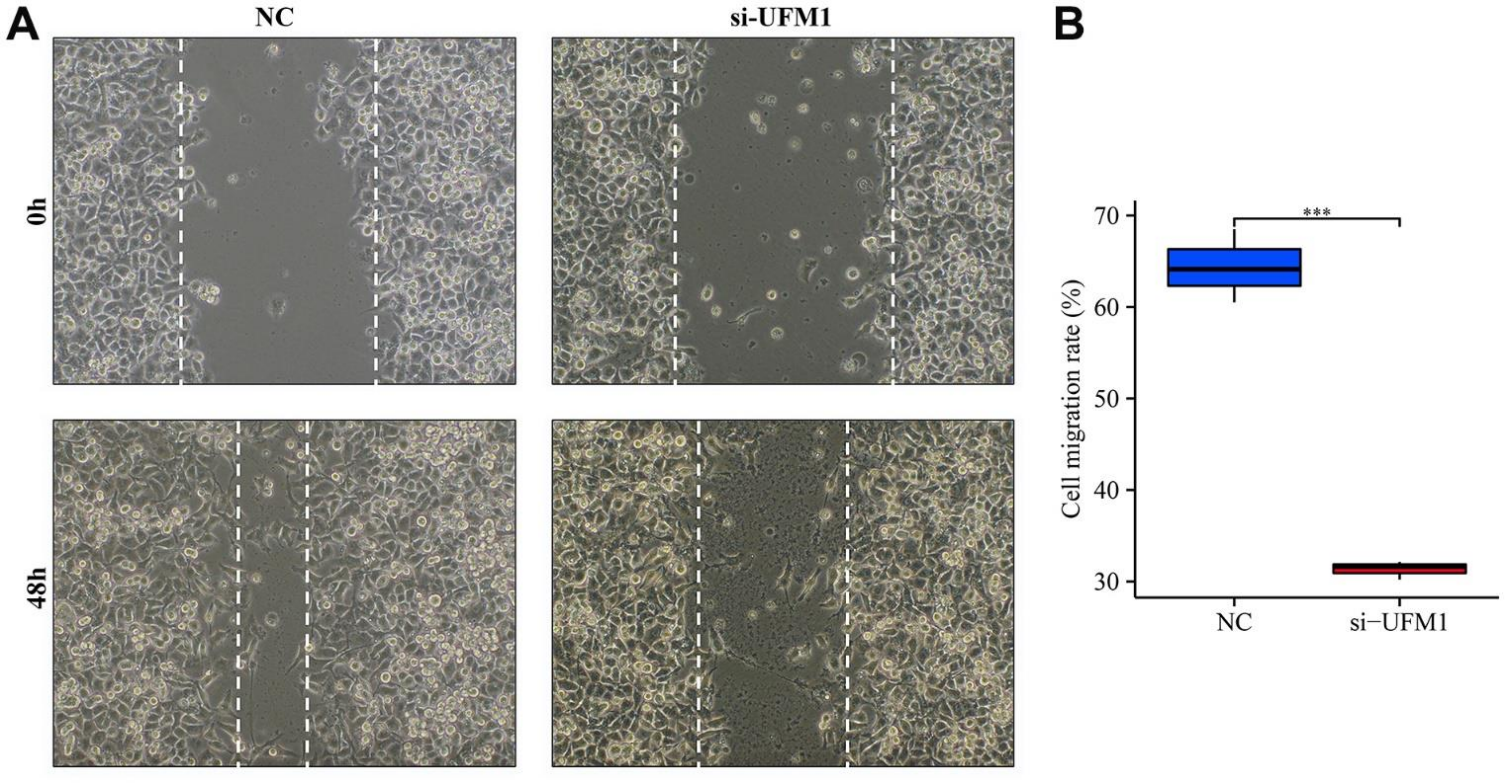
**Inhibition of UFM1 expression significantly reduces cell migration.** (**A**) Wound healing of cancer cells; (**B**) Cell migration rate in si-UFM1 vs. NC groups.

The mRNA and protein expression levels of UFM1 in the si-UFM1 group were significantly decreased using RT-PCR and Western blotting (Figure 9A–9C). CCK-8 detection exposed that inhibition of UFM1 expression significantly inhibited the cal27 cell proliferation, with statistically significant results (Figure 9D). The migration and invasion ability of cal27 cells were significantly decreased when UFM1 expression was inhibited (Figures 9E, 10).

### UFM1 expression levels correlated with ubiquitination and immune cell infiltration in OSCC

**Figure 11:**
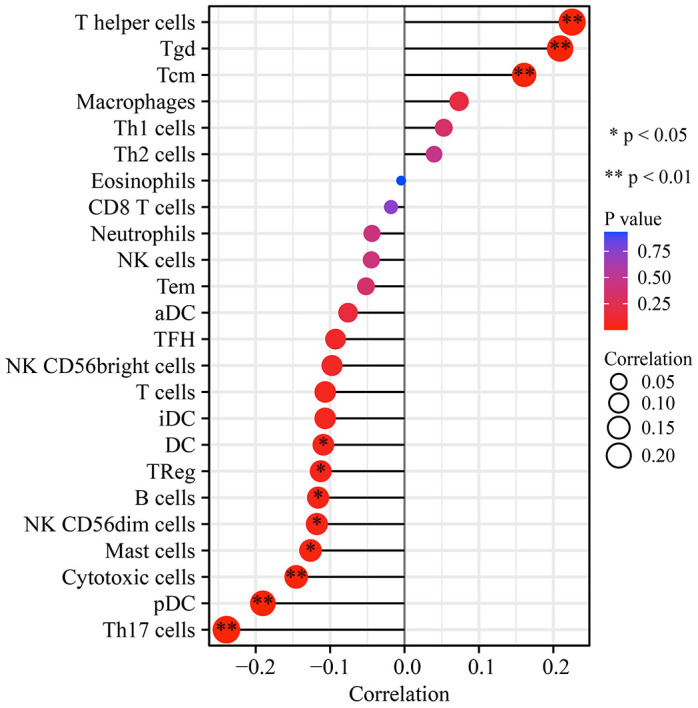
**UFM1 expression correlation with immune infiltrating cells in OSCC using bubble plot.** Note: OSCC, oral squamous cell carcinoma.

**Figure 12:**
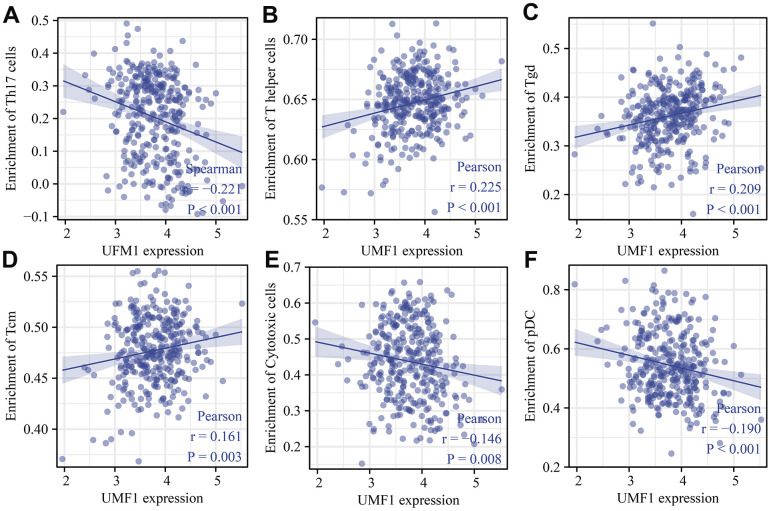
**UFM1 expression correlated with immune infiltrating cells in OSCC using scatter plot.** (**A**) Th17 cells; (**B**) T helper cells; (**C**) Tgd; (**D**) Tcm; (**E**) Cytotoxic cells; (**F**) pDC. Note: OSCC, oral squamous cell carcinoma.

Twenty-nine histone ubiquitination genes were obtained from the GSEA (Table 6). We found that the expression levels of UFM1 were significantly associated with the levels of ubiquitinated genes CDC73, RNF2, BMI1, RAG1, PCGF3, PCGF6, TRIM37, PAF1, CTR9, RYBP, CUL4B, PCGF1, PCGF2, UBE2E1, RNF40, WAC, UHRF1, and PCGF5 (Table 6). In addition, UFM1 expression levels significantly correlated with the Th17 cells, T helper cells, Tgd, pDC, Tcm, cytotoxic cells, mast cells, NK CD56dim cells, B cells, TReg, and DC levels in OSCC using bubble and scatter plots (Figures 11, 12).

**Table 6:** UFM1 expression levels correlated with ubiquitination in OSCC.

**Gene**	**Cor**	**P**	**Gene**	**Cor**	**P**
PCGF3	0.275	<0.001	RING1	-0.011	0.844
LEO1	0.064	0.245	RNF2	0.364	<0.001
DTX3L	0.090	0.102	BMI1	0.324	<0.001
DDB1	0.096	0.081	SKP1	0.008	0.885
DDB2	0.020	0.719	UBE2E1	0.175	0.001
RNF168	0.057	0.304	PCGF2	0.185	<0.001
RYBP	0.187	<0.001	CDC73	0.379	<0.001
UHRF1	0.122	0.026	PCGF6	0.252	<0.001
TRIM37	0.246	<0.001	PCGF5	0.121	0.029
WAC	0.131	0.017	CUL4B	0.186	<0.001
PAF1	-0.222	<0.001	KDM2B	0.033	0.551
BCOR	0.023	0.678	PCGF1	0.186	<0.001
RNF20	0.086	0.121	CTR9	0.205	<0.001
ATXN7L3	0.071	0.200	RNF40	0.161	0.003
RAG1	0.312	<0.001			

Note: OSCC, oral squamous cell carcinoma.

## DISCUSSION

OSCC is increasing annually and has become younger. The living habits of OSCC patients are affected greatly, and the prognosis is often poor. Studies have displayed that some biomarkers could predict the prognosis of OSCC patients, and inhibiting or promoting their expression could cause tumor growth arrest [2, 5, 14]. Several studies have reported the association of UFM1 with prognosis in GC, HCC, and breast cancer patients [7–10]. Currently, the role of UFM1 in OSCC has not been revealed. Our study found that UFM1 was overexpressed in unpaired and paired OSCC tissues. Cox regression and survival analyses revealed that UFM1 overexpression was significantly associated with shorter OS, DSS, and PFI in OSCC patients. The nomograms of T, N, and M stages and UFM1 expression levels were related to the prognosis of OSCC patients. In addition, inhibition of UFM1 expression could inhibit the proliferation, migration, and invasion of OSCC cells. It was discovered preliminarily that UFM1 overexpression was a risk factor for poor prognosis in OSCC patients and subsequently could be a potential biomarker for poor prognosis in OSCC patients.

Ubiquitination plays an important role in protein localization, metabolism, function, regulation and degradation, and in the cell cycle, proliferation, apoptosis, differentiation, metastasis, gene expression, transcriptional regulation, and signaling transmission in cancer [15–18]. UFM1 is a ubiquitin-like protein, and can bind to the target proteins UBA5 (E1), UFC1 (E2), and UFL1 (E3) through a three-step enzyme system. The protein modification of UFM1 can be reversed by UFM1 specific protease (UFSP), which in turn involves tumorigenesis [19]. We found that UFM1 is associated with the ubiquitinating genes: CDC73, RNF2, BMI1, RAG1, PCGF3, PCGF6, TRIM37, PAF1, CTR9, RYBP, CUL4B, PCGF1, PCGF2, UBE2E1, RNF40, WAC, UHRF1, and PCGF5 levels. Studies have depicted that RNF2, BMI1, TRIM37, and other ubiquitinating genes were related to cancer progression [20–24]. These results further suggested that the ubiquitin-like protein UFM1 has an important role in OSCC progression.

Tumor heterogeneity, immune status, and interrelationships between tumor and stromal cells within the tumor microenvironment might influence therapeutic efficacy [25]. A disturbed immune microenvironment was significantly associated with the progression of OSCC [25–27]. For example, Lenouvel et al. found that PD-L1 expression levels were associated with poor DSS and disease-free survival in OSCC patients. PD-L1 overexpression was positively associated with females, non-smokers, non-smokers and drinkers, advanced tumors, and high levels of PD-1, CD4^+^ and CD8^+^ [28]. Anti-PD-1 therapy improved the prognosis of patients with OSCC [29]. Our study found that the expression levels of UFM1 were significantly correlated with the levels of immune cells (Th17 cells, T helper cells, Tgd, pDC, Tcm, cytotoxic cells, mast cells, NK CD56dim cells, B cells, TReg, and DC), and immune cell markers, indicating that UFM1 might be related to immunity and the members of immune microenvironment homeostasis.

The roles of the UFM1 gene in OSCC were explored in this study via the high-quality samples from the available databases and basic research, deeming it reliable. However, this study had some limitations. First, the expression and prognostic values of UFM1 in OSCC, and the relationship between UFM1 expression and immune infiltration in OSCC were found using bioinformatics analysis, necessitating the collection of tissue samples from our hospital to verify in the future. Second, we must explore the roles and signaling mechanisms of UFM1 involved in the occurrence and development of OSCC *in vivo* and *in vitro*. In general, the results demonstrated that UFM1 was overexpressed in OSCC. The increased expression of UFM1 was associated with poor prognosis and immunity in OSCC patients. Inhibition of UFM1 expression could delay the progress of OSCC, indicating that UFM1 has the potential to become a prognostic biomarker and therapeutic target for the prognosis of OSCC patients for OSCC patients.

## CONCLUSIONS

Elevated UFM1 expression was associated with poor prognosis and immune infiltration in OSCC. Inhibition of UFM1 expression could inhibit the proliferation, migration, and invasion of OSCC cells. UFM1 could be a biomarker for prognosis and treating OSCC patients.
